# Spatial memory—a unique window into healthy and pathological aging

**DOI:** 10.3389/fnagi.2014.00035

**Published:** 2014-03-07

**Authors:** Thomas Wolbers, Paul A. Dudchenko, Emma R. Wood

**Affiliations:** ^1^Aging and Cognition Research Group, German Center for Neurodegenerative Diseases (DZNE)Magdeburg, Germany; ^2^School of Natural Sciences, University of StirlingStirling, UK; ^3^School of Biomedical Sciences, University of EdinburghEdinburgh, UK

**Keywords:** spatial navigation, aging, dementia, neuroscience, animal models, humans

The world's population is aging at an unprecedented rate, because the number of people aged over 60 will rise from 784 million in 2011 to 2 billion by 2050. Such a dramatic increase has made age-related cognitive decline and Alzheimer's disease (AD) a pressing social and health concern. Work described in this volume considers scientific efforts to understand the neural mechanisms of age-related changes in spatial navigation, both in humans and non-human animal models. A central theme in these papers is that damage to structures of the medial temporal lobe, including the hippocampus, contributes to the difficulties in spatial memory found in aging and AD.

Tanila (2012) describes the use of the Morris Water Maze (MWM) task as a tool for demonstrating memory impairments in mouse models of AD. Tanila (2012) points out important challenges in working with mice compared to rats—the former are prone to hypothermia, and exhibit strain differences in learning capacity. Strikingly, an impairment on the swim task is seen in all established models of AD in mice, though its relationship to the onset of amyloid plaque deposition or tau aggregation varies.

In the MWM, Yau and Seckl (2012) note that older rats with impaired performance have higher corticosterone levels than those who are unimpaired. Higher corticosterone levels shift the balance between mineralcorticoid- and glucocorticoid-receptor activation, and are associated with decreases in long-term potentiation and memory. Yau and Seckl (2012) review findings which show that reduction of an enzyme that increases glucocorticoids results in improved spatial memory in aged rodents.

Holden and Gilbert (2012) review studies which show that pattern separation abilities are impaired in older humans, monkeys, and rodents. Such a capacity likely relies on the hippocampus, and in particular the dentate gyrus/CA3 cell regions. They hypothesize that impaired pattern separation may result in impaired episodic memory in aging.

Penner and Mizumori (2012) also relate the pattern separation function of the hippocampus in terms of the recognition of contexts. Their proposal is that the hippocampus produces an error signal when a context is unexpected, and this ultimately drives dopaminergic neurons in the ventral tegmental area. Aging affects this circuit by altering hippocampal representations of context, mesolimbic-ventral striatum interactions, and the dopaminergic system.

Turning to humans, Adamo et al. (2012) investigated how aging affects path integration, a key navigational process. Both task complexity and the sources of information available to participants (i.e., visual vs. vestibular) had a substantial impact on the results. These findings have important methodological implications, because studies on spatial navigation are often confined to one sensory modality and do not systematically manipulate task complexity.

Several studies demonstrate deficits in allocentric processing in healthy older adults. Rosenbaum et al. (2012) showed that memory for the layout of long familiar Toronto landmarks did not differ dramatically between young and older participants, but the latter made many more errors in learning a new route in a hospital. These results support a model where episodic-like representations of spatial information (hippocampus dependent) give rise to more schematic (less detailed, but hippocampus independent) representations with repeated experience.

Using virtual environment (VE) technology, Yamamoto and DeGirolamo (2012) found that older participants had difficulties reconstructing the layouts of landmarks encountered in a virtual city. Interestingly, performance was not impaired when they experienced the environments from a bird's eye perspective. These results suggest that spatial learning through exploratory navigation may be particularly vulnerable to adverse effects of aging, whereas elderly adults may be able to maintain their map reading skills relatively well.

Wiener et al. (2012) had participants learn a route through a VE that contained multiple intersections. Compared to young controls, older adults had greater problems during route retracing than during route repetition. While route repetition can be solved with egocentric response or route strategies, successfully retracing a route requires allocentric processing. These age-related deficits in route retracing are discussed in the context of a potential shift from allocentric to egocentric navigation strategies as a consequence of age-related hippocampal degeneration.

A bias toward egocentric response strategies with increasing age was also observed by Bohbot et al. (2012). A virtual 8-arm radial maze served to assess spontaneous navigation strategies, i.e., hippocampal-dependent spatial strategies vs. caudate nucleus-dependent response strategies. Results showed that from childhood to old age, the spontaneous use of egocentric response strategies increased substantially. In a related study, Konishi and Bohbot (2013) showed that spontaneous spatial memory strategies positively correlated with gray matter density in the hippocampus of older participants. The combined results from both studies indicate that people who prefer to use spatial memory strategies in their everyday lives may have increased gray matter in the hippocampus and enhance the probability of healthy aging.

Beyond the hippocampus, aging also affects the integrity of a larger network of brain structures, including prefrontal cortex. Harris et al. (2012) found that older humans were impaired at switching from a route strategy to a place strategy on a virtual plus maze task. Interestingly, this did not reflect a general difficulty in switching between spatial strategies, as the switch from a place strategy to a route strategy was not impaired. This may imply that interactions between the prefrontal cortex and the hippocampus are affected with advanced age.

Finally, Pengas et al. (2012) demonstrate that spatial navigation impairments in AD relate to damage across a network, which offers complimentary lesion evidence to studies in healthy volunteers for the neural basis of topographical memory. Critically, the results emphasize that structures beyond the medial temporal lobe contribute to memory impairment in AD, which argues against common models in which memory impairment in AD is taken as a synonym for hippocampal degeneration.

The book concludes with a review of human aging and spatial navigation tasks by Gazova et al. (2012). They suggest that such navigation tasks may be a useful tool for identifying individuals who will go on to develop AD. Given the growing number of studies indicating that damage to the medial temporal lobe (including the hippocampus) is associated with wayfinding difficulties, Gazova et al. (2012) argue that the use of such spatial tasks may help to identify AD early in its course.
